# Statistical Difference Representation-Based Transformer for Heterogeneous Change Detection

**DOI:** 10.3390/s25123740

**Published:** 2025-06-15

**Authors:** Xinhui Cao, Minggang Dong, Xingping Liu, Jiaming Gong, Hanhong Zheng

**Affiliations:** 1 School of Artificial Intelligence, Guangzhou Huashang College, Guangzhou 511300, China; hscao9@gdhsc.edu.cn (X.C.); hsgong9616@gdhsc.edu.cn (J.G.); 2 Graduate School, St. Paul University Philippines Tuguegarao, Cagayan 3500, Philippines; 3 College of Information Science and Engineering, Guilin University of Technology, Guilin 541004, China; mgdong@glut.edu.cn; 4 School of Mathematics and Computer Science, Yan’an University, Yan’an 716000, China; lxp@yau.edu.cn; 5 Aerospace Information Research Institute, Chinese Academy of Sciences, Beijing 100094, China

**Keywords:** heterogeneous change detection, remote sensing images, transformer, statistical difference, structural similarity

## Abstract

Heterogeneous change detection refers to using image data from different sensors or modalities to detect change information in the same region by comparing images of the same region at different time periods. In recent years, methods based on deep learning and domain adaptation have become mainstream, which can effectively improve the accuracy and robustness of heterogeneous image change detection through feature alignment and multimodal data fusion. However, a lack of credible labels has stopped most current learning-based heterogeneous change detection methods from being put into application. To overcome this limitation, a weakly supervised heterogeneous change detection framework with a structure similarity-guided sample generating ($S^{3}$$G^{2}$) strategy is proposed, which employs differential structure similarity to acquire prior information for iteratively generating reliable pseudo-labels. Moreover, a Statistical Difference representation Transformer (SDFormer) is proposed to lower the influence of modality difference between bitemporal heterogeneous imagery and better extract relevant change information. Extensive experiments have been carried out to fully investigate the influences of inner manual parameters and compare them with state-of-the-art methods in several public heterogeneous change detection data sets. The experimental results indicate that the proposed methods have shown competitive performance.

## 1. Introduction

Change detection (CD) leverages multi-temporal remote sensing data with image processing and pattern recognition methods to extract change information, quantitatively analyze to determine the characteristics and processes of surface changes, and identify changes in surface objects or phenomena in time by comparing remote sensing images from different periods [1,2,3], which has been applied in urban planning [4], land resource supervision [5], disaster monitoring [6,7], and emergency response [8].

Traditional CD techniques predominantly hinge on the comparative analysis of multi-temporal remote sensing imagery to discern change information, which is achieved with the construction of difference images, feature transformation, and the application of unsupervised or supervised classification methodologies [9,10,11]. While these approaches have been extensively utilized in the realm of low- and medium-resolution imagery, they are fraught with several limitations [12,13]. Notably, they are characterized by complex procedural steps, a low degree of automation, and stringent requirements regarding data quality and scene complexity [3,14]. Furthermore, the efficacy in handling high-resolution and intricate scenes is notably constrained [15]. Conversely, deep learning-based CD techniques leverage sophisticated models such as convolutional neural networks (CNNs) and Transformers to automatically extract intricate, non-linear, and multi-scale features from multi-temporal remote sensing images, thereby achieving enhanced performance in CD [16,17,18]. Utilizing an end-to-end learning framework, these approaches are adept at managing change detection tasks within high-resolution and complex scenarios, thereby markedly elevating the precision and robustness of the detection process [19].

However, the majority of existing research in this domain is predicated on single-modality approaches. Single-modal CD relies on a single type of remote sensing data, which has drawbacks such as data limitations, susceptibility to noise, limited feature extraction capability, restricted applicability, lack of robustness, and difficulty in handling multitasking requirements, which limit its application effectiveness and detection accuracy in complex scenes [20,21,22]. Compared to single-modal CD, Heterogeneous CD (HCD), also known as multimodal CD, overcomes the limitations of single-modality data by integrating multiple remote sensing data sources [23,24]. HCD is particularly effective in handling change detection tasks in complex environments, thereby enhancing the capacity for feature learning and generalization [25]. Moreover, it reduces the impact of noise and error, and is better equipped to meet the demands of complex scenes and multitasking scenarios [14,26]. Consequently, HCD demonstrates substantial application value in remote sensing image CD [27].

Overall, the current challenges in HCD are primarily characterized by the significant intermodal disparities that complicate feature extraction and fusion, the scarcity of labeled data that constrains model training, the difficulty in accurately detecting change information within complex scenes, the need to balance computational resources with efficiency, and the insufficient generalization capability of existing models [28,29]. The contemporary research landscape of HCD is characterized by the increasing adoption of deep learning and domain adaptation techniques, which have facilitated the development of methods centered on feature alignment and multimodal data fusion [30,31]. These advancements have demonstrably enhanced the accuracy and robustness of change detection by mitigating the disparities between different modalities [32]. Despite these progressions, HCD continues to confront substantial challenges. These include the complexities associated with feature extraction and fusion due to significant intermodal differences, the limitations imposed by the scarcity of labeled training data, the intricacies of accurately identifying change information within complex scenes, the need to optimize the balance between computational resources and efficiency, and the persistent issue of insufficient model generalization capability [33,34,35]. To address these multifaceted challenges, researchers are actively engaged in exploring weakly supervised and unsupervised learning paradigms, devising more efficient data fusion strategies, and developing novel approaches to bolster model robustness and generalization ability [21]. These efforts are aimed at driving the continued evolution and refinement of HCD technologies.

However, HCD involves using image data from different sensors or different modalities to detect change information in the same region. However, significant differences between modalities (e.g., optical vs. radar images) can severely affect the effectiveness of feature extraction and fusion, leading to a decrease in the accuracy and robustness of change detection. Completely unsupervised methods can alleviate this problem, but they often fail to meet the diverse scene requirements and are susceptible to the quality of pseudo-labeling. While the existing HCD methods [36,37,38] show obvious limitations when dealing with complex scenes, such as sensitivity to noise, limited feature extraction capability, and insufficient generalization ability.

In this paper, to address the problems of large modal differences, scarcity of labeled data, and insufficient detection accuracy in complex scenarios in HCD, a heterogeneous change detection framework based on weakly supervised learning is proposed, which combines a Structural Similarity-Guided Sample Generation strategy ($S^{3}$$G^{2}$) and a Statistical Difference representation Transformer (SDFormer). The framework generates reliable pseudo-labeled samples iteratively to expand the training set, and takes advantage of the Transformer architecture to reduce the influence of modal differences and enhance the extraction of change information, thus significantly improving the accuracy and robustness of heterogeneity change detection while optimizing the utilization efficiency of computational resources. Overall, the main contributions of this paper are as follows:

(1) The Statistical Difference representation Transformer (SDFormer) is proposed to reduce modal differences and enhance the ability of change information extraction through feature-level statistical analysis.
(2) A weakly supervised framework combined with structural similarity-guided sample generation strategy ($S^{3}$$G^{2}$) is designed to iteratively generate reliable pseudo-labels to expand the training set and improve the model performance.
(3) A statistical difference tokenization scheme within the Transformer architecture is developed to explicitly mitigate modality discrepancies while leveraging global contextual awareness, enhancing accuracy and robustness in complex HCD scenarios.

The remainder of this article is structured as follows. Section 2 describes the related work. Section 3 introduces the proposed method. Results and discussion are presented in Section 4 and Section 5, respectively. Section 6 concludes this paper.

## 2. Related Works

Heterogeneous change detection (HCD) refers to the use of data from different modalities (e.g., different sensors, data sources, or types of information) for change analysis [39,40,41]. In HCD, the aim is to improve the accuracy and robustness by combining information from multiple data sources [42]. The features and data of different modalities are usually complementary, so combining multimodal information can help overcome the limitations of a single data source, such as noise, low resolution, and missing information [43,44].

Graphics-based HCD combines computer graphics techniques and multimodal data analysis methods to identify and analyze changes in images, involving geometric modeling, reconstruction, and texture mapping [43,45,46]. Sun et al. [47] proposed an Improved Non-Local Patch-based Graph (INLPG) method for unsupervised HCD, which detects changes by comparing the structure of the image rather than the pixel values, which is robust and applicable to a wide range of change detection scenarios, including CD in homogeneous and heterogeneous remote sensing images. In [48], an Iterative Robust Graph and Markovian co-Segmentation method (IRG-McS) is designed, which exploits the self-similarity of images to construct robust K-nearest-neighbor graphs to represent the structure of each image, and compares these graphs by graph mapping to compute forward and backward difference images to avoid the confusion of heterogeneous data. In addition, IRG-McS [48] fuses the forward and backward difference images for change detection via Markov co-segmentation model and feeds back the change regions into the graph construction process. In [49], a Sparse-Constrained Adaptive Structure Consistency-based method (SCASC) is proposed to represent the structure of the pre-event image by constructing an adaptive similarity map, utilize the structural consistency to map the pre-event image into the domain of the post-event image, and compute the difference image and employ the super-pixel-based Markov Random Field Segmentation model to segment the difference image into changed and unchanged categories. Moreover, Sun et al. [50] proposed an unsupervised image regression method based on structural graphs (called GIR-MRF) to capture local and global structural information by learning the structural graphs of images and using structural consistency to map pre-event images to post-event image domains, introducing global self-expression constraints and local similarity constraints, as well as a Markov Random Field Segmentation model combining the change information and the spatial information, thus realizing heterogeneous CD. Moreover, a Locality-Preserving Energy Model (LPEM) [51] is designed to generate change maps by leveraging modality-invariant topological relationships between super-pixel pairs and enforcing structural consistency in feature space and label continuity in geographic space, without intermediate difference image computation. Han et al. [52] proposed an improved training method with Hierarchical Extreme Learning Machine (HELM), which addresses challenges like noise sensitivity and manual sample selection by introducing automatic training sample extraction, image smoothing for noise reduction, and hierarchical feature learning.

Deep learning-based HCD improves the accuracy and robustness due to the powerful expressive capability that enables it to automatically learn useful features from different modal data and efficiently perform multimodal data fusion [12,14]. Liu et al. [53] proposed an unsupervised Symmetric Convolutional Coupling Network (SCCN) for heterogeneous optical and radar images, which computes a disparity map and enables change detection by transforming the input images into a feature space with more consistent feature representations. In [54], two deep convolutional neural network-based architectures (X-Net and ACE-Net) are proposed. X-Net [54] consists of an architecture of two fully convolutional networks, each of which is separately responsible for mapping data from one domain to another. Specifically, one network maps data from the optical image domain to the Synthetic Aperture Radar (SAR) image domain, and the other performs the opposite mapping. X-Net [54] is trained with weighted translation loss and cyclic consistency loss to ensure that the mapping is as accurate as possible in the invariant region, while not enforcing mapping consistency in the changing region. ACE-Net [54] introduces a latent space as an intermediate representation between the two domains. ACE-Net [54] consists of two autoencoders, each consisting of an encoder and a decoder, which are used to map the data from the original domain to the latent space, and from the latent space back to the original domain. In addition, ACE-Net includes a discriminator for adversarial training to ensure that the code spaces of the two autoencoders in the potential space are aligned. Wu et al. [55] proposed an unsupervised Commonality Autoencoder CD method (CACD), which extracts features by means of a convolutional autoencoder, converts the representation of one heterogeneous image to that of another, and is able to efficiently identify the common features of unchanged regions and generate a difference map for change detection. Chen et al. [21] proposed an unsupervised HCD method, which represents the structural information of a multimodal image by constructing a structural graph and learns the structural information in the graph with a Structural Relation Graph Convolutional AutoEncoder (SR-GCAE) to measure the similarity between local and non-local structural relations. Difference images are generated by calculating the similarity levels of the two structural relations, an adaptive fusion strategy is used to fuse these difference images, and finally, morphological filtering post-processing is used to optimize the detection results. In [56], the content cleansing network is proposed to mitigate pseudo-changes in cross-platform and multi-temporal VHR images by decoupling content-style features with multi-resolution encoding and image restoration. Han et al. [57] designed an unsupervised graph autoencoder for multimodal change detection by compensating structural differences, trained via reconstruction–sparsity–structural losses. Recently, Liu et al. proposed a commonality feature representation learning (CFRL) for HCD [24], and achieved the current state-of-the-art performance. These approaches have advanced the progress of heterogeneous CDs.

In terms of Transformer-based architecture, Liu et al. [58] integrates digital surface model via cross-attention, enforces multitask consistency between semantic changes and height-derived pseudo-changes with soft-thresholding constraints. Moreover, Zou et al. [59] proposed an improved multi-scale and spectral-wise Transformer for hyperspectral image change detection, integrating a multi-scale feature fusion module, locality self-attention to mitigate spectral embedding challenges. In [60], a novel two-stream attention-in-attention vision architecture is proposed, called SimPool, which integrates SimPool and ResMLP.

While existing HCD methods have advanced capabilities, critical limitations remain unaddressed. Graph-based approaches such as IRG-McS [48] and GIR-MRF [50], while effective in capturing structural consistency, rely on handcrafted similarity metrics that struggle to adapt to non-linear feature interactions inherent in complex multimodal data. Deep learning methods like CACD [55], AEKAN [23], and CFRL [24] demonstrate enhanced modality-independent feature learning capabilities between heterogeneous features, but are still severely limited by the limitation that the lack of reliable supervision information leads to unstable learning of the methods. Though Transformer-based architectures [58] show promise in modeling global contextual relationships, their pseudo-label generation strategies lack structural guidance, leading to error propagation during iterative training, while their feature fusion mechanisms insufficiently address statistical distribution discrepancies between heterogeneous modalities. This analysis reveals three interconnected gaps: the absence of frameworks that jointly address feature-level statistical divergence and annotation scarcity, the underutilization of differential structural consistency as prior knowledge in pseudo-label refinement, and the limited exploitation of Transformers for cross-modal statistical alignment. These insights directly inform our dual innovations—the $S^{3}$$G^{2}$ strategy leverages differential structural similarity to iteratively refine reliable pseudo-labels under sparse supervision, while SDFormer’s statistical difference tokenization enables Transformer-driven harmonization of cross-modal representations, thereby simultaneously resolving modality gaps, label scarcity, and complex scene challenges through unified statistical–geometric learning.

## 3. Methodology

### 3.1. Overview

Benefiting from the outstanding ability of change information extraction, deep learning-based techniques and methods have been widely applied in change detection tasks [16]. Based on sufficient supervised learning, these data-driven deep neural networks can effectively identify and annotate changed areas according to the need of change detection applications [61]. However, these supervised deep learning CD methods usually rely on massive labeled data to acquire acceptable CD ability. To overcome this limitation, unsupervised deep learning-based frameworks are invented to face the situations with limited training samples. In order to overcome this limitation, unsupervised deep learning frameworks are proposed to deal with the limited training samples [62], which is often the case in heterogeneous image change detection applications.

**Figure 1:**
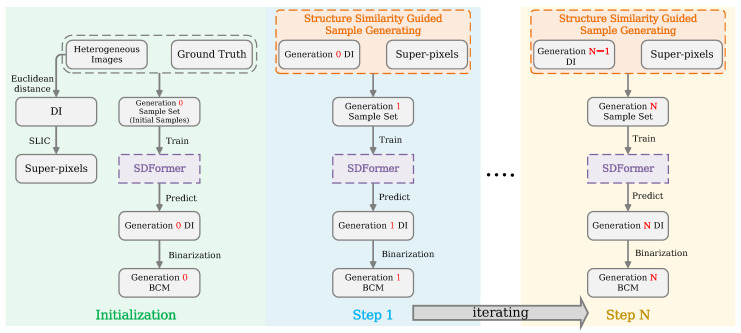
Overview of the proposed bitemporal heterogeneous imagery change detection framework.

However, totally unsupervised frameworks sometimes cannot fulfill varied CD requirements and image modalities in HCD applications, since most of them possess fixed strategies to generate pseudo-training-samples or directly detect change information. Based on these facts, a weakly-supervised HCD framework with a Statistical Difference representation Transformer (SDFormer) is proposed, which utilizes a limited number of labeled data and differential structure similarity to iteratively generate training samples and deal with varied HCD scenes with acceptable accuracy. The proposed heterogeneous image change detection framework can be demonstrated as two main stages, initialization and iteration, as shown in Figure 1.

### 3.2. Initialization

In this stage, one percentage of pixels from current bitemporal heterogeneous imagery are randomly selected and labeled for the first epoch of training, which build initial HCD ability for the proposed SDFormer, thus facilitating the following structure similarity-guided sample generating ($S^{3}$$G^{2}$) strategy in the iteration stage. In the practical application, initial training samples, i.e., generation 0 sample set, can be manually labeled to fit varied change detection requirements. After the initial training samples gained, they are utilized to train the proposed SDFormer for several rounds until the training loss stops dropping. Considering that heterogeneous imagery usually has varied patterns and modalities, the differential information is directly used to extract structure similarity for pseudo-sample generating. To achieve this goal, the Euclidean distance is firstly used to directly measure pixel-wise change intensity between bitemporal heterogeneous images as shown below:

(1) $${DI}_{i,j}=∥{T1}_{i,j}-{T2}_{i,j}∥$$

where ${DI}_{i,j}$, ${T1}_{i,j}$, and ${T2}_{i,j}$ indicate each pixel of difference intensity map and corresponding bitemporal heterogeneous imagery, respectively, and ∥·∥ is the vector-wise Euclidean distance. Afterwards, the Simple Linear Iterative Clustering (SLIC) is employed to acquire the super-pixel map according to the difference intensity map, which uses amorphous homogeneous regions to describe the spatial areas sharing similar differential pattern, thus extracting differential structure similarity. To sum up, at the end of initialization stage, a super-pixel map carrying differential structure similarity, an initially trained SDFormer, and corresponding raw prediction are acquired, which will be utilized to expand the scale of sample set for better change detection performance in the following iteration stage.

### 3.3. Iteration

At the iteration stage, the proposed structure similarity-guided sample generating strategy is firstly applied to generate pseudo-samples based on the similarity of differential patterns and the existing initial samples with ground truth, in order to expand the size of the training set. Subsequently, the enlarged sample set with both genuine and pseudo-labels will be used to train the proposed SDFormer, resulting in better HCD performance. After each iteration, as the number of samples increases, the network performance will also improve accordingly, if there is a rational sample generating strategy.

#### Structure Similarity-Guided Sample Generating

**Figure 2:**
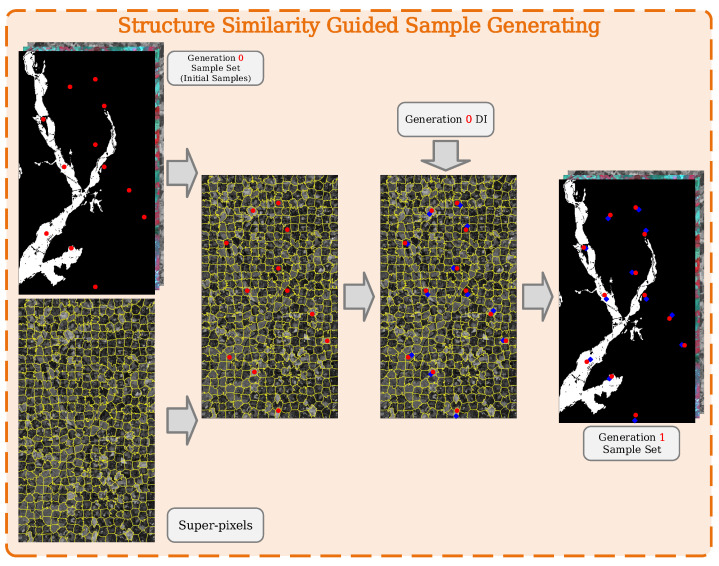
Processing of the proposed structure similarity-guided sample generating. Note: Note: The red dots represent the initial sample points, and the blue dots represent the newly added samples.

A large number of weakly supervised and unsupervised change detection methods rely on reliable iterative pseudo-label-generating strategies to gain stable performance improvement, since poison pseudo-label will downgrade the model performance after training. To generate credible pseudo-samples, the super-pixels is firstly utilized to describe similar change patterns, since similar change categories lead to consistent difference intensities, which can be captured by SLIC algorithm and clustered into super-pixels. Combining the super-pixels with ground truth in the initial samples, the proposed $S^{3}$$G^{2}$ strategy can be informed with prior change information. That is, by identifying which super-pixel each sample belongs to, it can be preliminarily determined that the pixels contained in that super pixel are highly likely to have the same label as the sample. However, considering that super pixels cannot be clustered completely correct, the difference intensity map brought by the raw prediction of SDFormer is employed to find and annotate the pixel with the highest credibility within the super-pixel. After each initial sample point is queried and processed, the size of the sample set will double, thus helping the proposed SDFormer reach higher performance (see Figure 2).

### 3.4. Statistical Difference Representation Transformer

**Figure 3:**
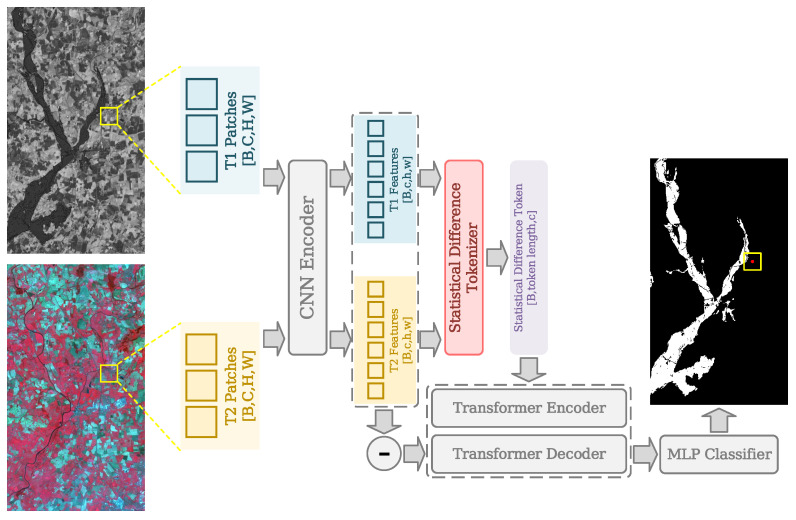
Overview of the proposed SDFormer. Note: “−” refers to the subtraction operation.

Transformer models have been widely used in remote sensing fields [15]. Compared to conventional CNN models, Transformers usually extract features in a different way with richer context and global information, which can better fit the HCD requirements, since this can lower the influence brought by difference in modality, thus improving the recognition for relevant changes in bitemporal heterogeneous imagery [63]. In the proposed SDFormer, the influence of modality differences is further reduced by leveraging feature-level statistical analysis, capitalizing on the advantages of Transformers, as shown in Figure 3.

The proposed SDFormer can be divided into four different parts, i.e., a CNN encoder, a statistical difference tokenizer, a Transformer encoder and decoder, and a Multi-Layer Perceptron (MLP), for better introduction. Firstly, bitemporal heterogeneous image patches with the spatial size of 15 × 15 are input into a Siamese CNN encoder with one convolutional layer with the kernel size of 7 × 7 and two convolutional layers with the kernel size of 3 × 3, which project heterogeneous imagery to a common feature space for better change extraction. All these convolutional layers have batch normalization layers and rectified linear units to better extract bitemporal features. This process is demonstrated as follows:

(2) $$f_{1}={\mathrm{Encoder}}_{\mathrm{CNN}}\left(i_{1}\right)$$

(3) $$f_{2}={\mathrm{Encoder}}_{\mathrm{CNN}}\left(i_{2}\right)$$

where $f_{1}\in R^{B\times C\times H\times W}$, $f_{2}\in R^{B\times C\times H\times W}$, and $i_{1}$, $i_{2}$ represent bitemporal heterogeneous feature maps and image patches, respectively, and B, C, H, and W indicate the batch size, channel size, height, and width of feature maps, respectively. To better enhance valid change information and lower the influence of modality gap between bitemporal heterogeneous features, the feature-level statistical analysis is employed, the statistical difference tokenizer, to represent and tokenize the bitemporal features. More specifically, channel-wise maximum, minimum, average, and standard deviation are acquired as bitemporal heterogeneous statistical tokens. Then, the statistical difference token can be obtained by subtracting the bitemporal statistical tokens. The detailed process is represented as follows:

(4) $${\mathrm{token}}_{1}=\left[\max\left(f_{1}\right),\min\left(f_{1}\right),\mathrm{avg}\left(f_{1}\right),\mathrm{std}\left(f_{1}\right)\right]$$

(5) $${\mathrm{token}}_{2}=\left[\max\left(f_{2}\right),\min\left(f_{2}\right),\mathrm{avg}\left(f_{2}\right),\mathrm{std}\left(f_{2}\right)\right]$$

(6) $${\mathrm{token}}_{d}=\left|{\mathrm{token}}_{1}-{\mathrm{token}}_{2}\right|$$

where $\left[\cdot \right]$ and $\left|\cdot \right|$ are channel-wise concatenation and absolute value generator, respectively, and ${token}_{d}\in R^{B\times C\times 4}$ is the statistical difference token with less modality information and more valid change information.

Based on the success of Transformer models, the Transformer encoder and decoder with cross-attention mechanisms is employed to better detect valid changes between bitemporal heterogeneous imagery, which shares a similar structure with [15]. Different from the conventional bitemporal Transformers, statistical difference information is utilized to directly enhance and refine feature-level differential information rather than bitemporal features, which further lower the influence of modality difference, as shown below:

(7) $$f_{d}={\mathrm{Decoder}}_{\mathrm{transfomer}}\left(\left|f_{1}-f_{2}\right|,{\mathrm{Encoder}}_{\mathrm{transfomer}}\left({\mathrm{token}}_{d}\right)\right)$$

Finally, refined differential feature $f_{d}\in R^{B\times C\times H\times W}$ is processed by MLP to predict the annotation of current central pixel from the input bitemporal heterogeneous image patches. To sum up, the differential statistical information is leveraged in the proposed SDFormer to better represent valid change information and further get rid of the interference of modality difference between bitemporal heterogeneous imagery, thus improving the performance.

## 4. Experiments and Results

In this section, our experiments and results are presented on three public HCD data sets. First, experimental data sets are described in detail. Second, the comparison methods and evaluation metrics selected are provided in this experiment. Then, the deployment details of the proposed method and other comparison methods are given. Finally, the experimental results of the proposed approach with other comparison methods are compared and analyzed to verify its effectiveness and superiority.

### 4.1. Data Set Descriptions

**Figure 4:**
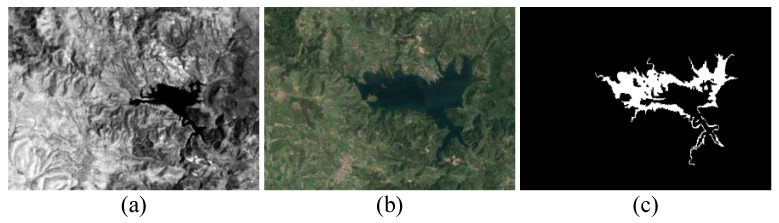
Data set #1: (**a**) T1 image; (**b**) T2 image; (**c**) ground truth image.

**Data Set #1:** As shown in Figure 4, data set #1 consists of a T1-time image from Landsat-5 and a T2-time from Google Earth. They were captured in Sardinia, Italy in September 1995 and July 1996, with sizes of 300×412×1 and 300×412×3, respectively. Their spatial resolution is 30 m. The change events described in this data are mainly lake expansion. The difficulty of this data set is that the spectral differences and modality differences are large, and it is challenging to detect fine change details.

**Figure 5:**
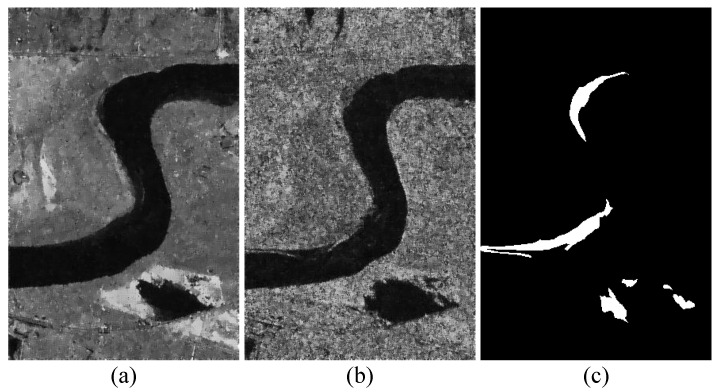
Data set #2: (**a**) T1 image; (**b**) T2 image; (**c**) ground truth image.

**Data Set #2:** The T1-time and T2-time images of data set #2 are composed of SAR and optical images acquired from Radarsat-2 and Google Earth, respectively, as presented in Figure 5. They were captured in June 2008 and September 2010 at the Yellow River, China respectively, with both sizes of 343×291×1. Their spatial resolution is 8 m. The change events depicted in this data are mainly embankment. The main challenge of this data set is the huge modal difference between optical images and SAR images, which makes it difficult to accurately detect changes because SAR images contain more coherent speckle noise than optical images.

**Figure 6:**
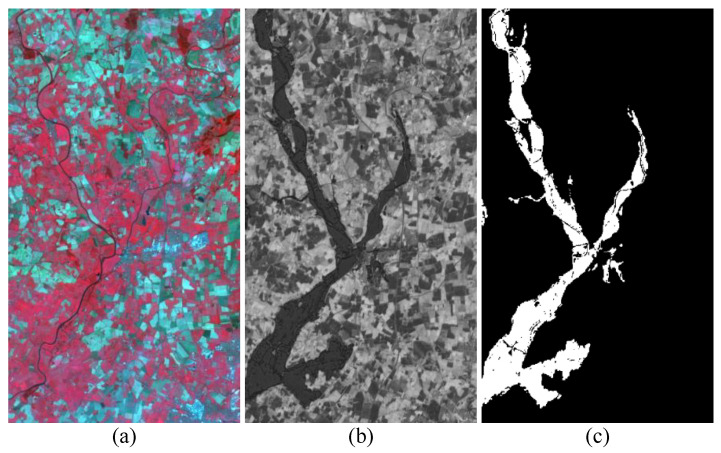
Data set #3: (**a**) T1 image; (**b**) T2 image; (**c**) ground truth image.

**Data Set #3:** As given in Figure 6, data set #3 consists of a T1-time image from Spot and a T2-time from NDVI. They were captured in 1999 and 2000 at Gloucester, UK, with sizes of 900×554×3 and 900×554×1, respectively. Their spatial resolution is about 25 m. Flooding is the main change event described in this data. The difficulty of this data set is similar to that of data set #1. There are huge spectral and modal differences, making it difficult to effectively identify changes through comparison.

### 4.2. Comparative Approaches and Evaluation Indicators

#### 4.2.1. Comparative Approaches

In the experiment, the popular and advanced traditional graph analysis methods are selected to compare with the proposed SDFormer method, including IRG-McS [48], SCASC [49], and GIR-MRF [50]. These methods are one of the current mainstream HCD methods and have achieved excellent detection performance on related tasks. Therefore, several convincing traditional graph analysis methods are selected for comparison. In addition, popular and latest deep learning-based methods are also selected to compare with the proposed method, including X-Net [54], AEC-Net [54], CACD [55], SR-GCAE [21], AEKAN [23], and CFRL [24]. These methods are representative methods among HCD methods based on deep learning. It is worth noting that AEKAN [23] and CFRL [24] are the latest methods and have achieved state-of-the-art (SOTA) performance on multiple data sets. Based on the superiority of these methods, it is meaningful to select these methods to compare with the proposed method.

#### 4.2.2. Evaluation Indicators

In our experiments, the popular evaluation metrics in HCD is adopted to calculate the quantitative accuracy of each method, namely, Overall Accuracy (OA), Kappa Coefficient (KC), and F1-Score (F1). Based on the binary confusion matrix, these metrics can be calculated as follows:

(8) $$\mathrm{OA}=\frac{\mathrm{TP}+\mathrm{TN}}{\mathrm{TP}+\mathrm{TN}+\mathrm{FP}+\mathrm{FN}},$$

(9) $$\mathrm{KC}=\frac{2\times \mathrm{PRE}\times \mathrm{REC}}{\mathrm{PRE}+\mathrm{REC}},$$

where

(10) $$\mathrm{PRE}=\frac{\mathrm{TP}}{\mathrm{TP}+\mathrm{FP}},$$

and

(11) $$\mathrm{REC}=\frac{\mathrm{TP}}{\mathrm{TP}+\mathrm{FN}}.$$

(12) $$\mathrm{KC}=\frac{\mathrm{OA}-p_{e}}{1-p_{e}},$$

where

(13) $$p_{e}=\frac{\left(\mathrm{TP}+\mathrm{FN})\times (\mathrm{TP}+\mathrm{FP})+(\mathrm{FP}+\mathrm{TN})\times (\mathrm{FN}+\mathrm{TN}\right)}{{\left(\mathrm{TP}+\mathrm{TN}+\mathrm{FP}+\mathrm{FN}\right)}^{2}},$$

where TP, TN, FP, and FN represent the True Positive (TP), True Negative (TN), False Positive (FP), and False Negative (FN) in the binary confusion matrix. These three evaluation indicators can comprehensively evaluate each approach.

### 4.3. Implementation Details

In the experiments, IRG-McS [48], SCASC [49], and GIR-MRF [50] followed the original settings of the paper. X-Net [54] and ACE-Net [54] could not be deployed due to low code versions, so the results of some comparison methods are obtained from other papers. For CACD [55], except for data set #1, the redeployed code is used to obtain the results for the rest of the data. In addition, SR-GCAE [21], AEKAN [23], and CFRL [24] were deployed using the original code and the best results were obtained through trial and error.

In the proposed SDFormer, the proposed method is deployed in the PyTorch 1.8 deep learning framework and trained using a NVIDIA 3090 Graphics Card (ASUS, Taiwan, China). Specifically, the Adam is used as the optimizer, the learning rate is set to 0.0001, and the weight decay is set to 0.00001. The batch size of the proposed method is set to 128. Notably, in the proposed framework, the iterative process terminates after 50 epochs for all experiments. In addition, in the comparative experiments, our parameter settings on the three data sets are set as follows: The number of super-pixels is 1000, the patch size is 15, and the ratio of training samples is 1%. Based on the above settings, the comparative experiments are constructed to verify the effectiveness and superiority of the proposed method. Notably, the number of parameters and computational cost of the proposed SDFormer are 3.3 M and 101.26 M FLOPs, respectively. The proposed method requires fewer computational resources, and no comparison of computational resources is provided in the comparison methods. Therefore, this experiment does not compare and discuss the computational cost of different methods.

### 4.4. Comparison of DIs with Different Methods

In order to verify the quality of the difference images (DIs) detected by different approaches, the Receiver Operating Characteristic (ROC) curve and Precision–Recall (PR) curve are first selected to compare and analyze the selected methods. Meanwhile, we also quantitatively compare the area under the ROC curve (AUR) and the area under the PR curve (AUP) of different methods to verify the advantages of the proposed approach. The experimental results and analysis on the three data sets are as follows.

#### 4.4.1. Comparison Based on ROC

**Figure 7:**
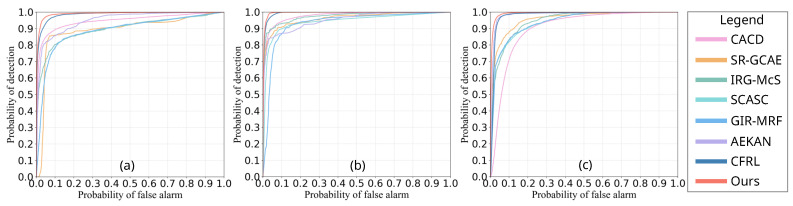
Comparison of ROC curves of different methods on the data sets #1–#3 for DIs: (**a**) data set #1, (**b**) data set #2, (**c**) data set #3.

Figure 7 shows the ROC curves of the proposed SDFormer and all compared methods on three data sets #1–#3. Overall, the proposed SDFormer method based on structural similarity-guided sample generation presents better DI performance. For data set #1, the curve of the proposed SDFormer is closer to the upper left corner, as shown in Figure 7a. The second closest to the upper left corner is CACD [55], and the gap with the proposed SDFormer is very significant. This shows that the DI of the proposed approach shows better separability; that is, it can achieve higher detection accuracy with fewer false detections. Similarly, for data set #2, the proposed SDFormer and CACD [55] obtained the best and second-best results, respectively. The difference is that the proposed SDFormer obtained a slightly better performance than AEKAN [23] on data set #3; that is, the degree of proximity of the two to the upper left corner is almost the same. In addition, as shown in Table 1, the area under the ROC curve (AUR) of the proposed SDFormer is significantly higher than that of other compared methods. The above results and analysis present that the proposed SDFormer has achieved competitive performance on all three data sets.

#### 4.4.2. Comparison Based on PR

**Figure 8:**
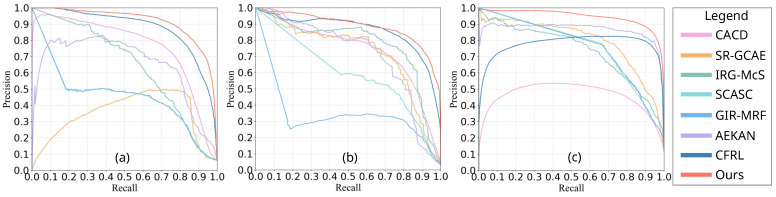
Comparison of PR curves of different methods on the data sets #1–#3 for DIs: (**a**) data set #1, (**b**) data set #2, (**c**) data set #3.

In addition to the comparison of ROC curves, the comparison of PR curves is depicted in Figure 8. For data set #1, the proposed SDFormer is closer to the upper right corner than all other compared methods. Compared with the proposed approach, CACD [55] and AEKAN [23] are significantly inferior. For data sets #2 and #3, the proposed SDFormer also achieved the best performance. IRG-McS [48] and AEKAN [23] achieve the second-best results on data sets #2 and #3, respectively. As presented in Table 1, the area under the PR curve (AUP) of the proposed SDFormer performs better than other methods. This is because the proposed SDFormer can represent the changes between heterogeneous images through statistical difference feature learning. At the same time, the proposed method uses the structural similarity sample generation strategy to effectively obtain reliable labeled samples to train our SDFormer. This shows that our SDFormer is more likely to obtain accurate and comprehensive detections than other methods, proving the reliability of our method in obtaining binary detections.

### 4.5. Comparison of BCIs with Different Methods

In this section, the binary change images (BCIs) of different comparison methods are analyzed on three data sets #1–#3 from the perspectives of quantitative and visual results to demonstrate the effectiveness and superiority of the proposed SDFormer. The results of each data are analyzed as follows.

#### 4.5.1. Results on Data Set #1

**Figure 9:**
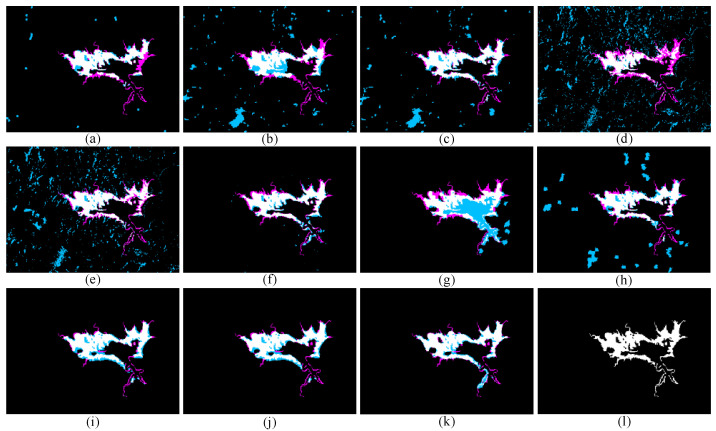
Binary change results of different methods on data set #1: (**a**) IRG-McS [48], (**b**) SCASC [49], (**c**) GIR-MRF [50], (**d**) X-Net [54], (**e**) ACE-Net [54], (**f**) CACD [55], (**g**) SR-GCAE [21], (**h**) AEKAN [23], (**i**) CFRL+Otsu [24], (**j**) CFRL+FLICM [24], (**k**) Ours, and (**l**) Ground truth image. Note: White and black represent TP pixels and TN pixels, respectively; sky blue and magenta represent FP and FN pixels, respectively.

The BCIs of the proposed SDFormer and seven compared methods are obtained. The comparisons of quantitative accuracy and visual BCIs are shown in Table 2 and Figure 9. From the quantitative comparison, the proposed SDFormer achieves the best accuracy, and CFRL [24] and CACD [55] achieve the second-best and third-best accuracy. For example, for CFRL [24], the proposed SDFormer achieves 0.005, 0.039, and 0.036 improvements in OA, KC, and F1, respectively. Compared with CACD [55], our SDFormer improves OA, KC, and F1 by 0.004, 0.048, and 0.045, respectively. Compared with the IRG-McS [48], our proposed SDFormer boosts by 0.008, 0.085, and 0.081 in terms of OA, KC, and F1, respectively; The reason is that the proposed method proposes a Transformer network based on statistical differences to learn the differences between heterogeneous images to relatively alleviate the impact of modality differences. At the same time, a reliable training sample is obtained by guiding the sample generation strategy through structural similarity to supervise the proposed SDFormer. This is conducive to improving the detection accuracy of HCD in a supervised manner. In addition to quantitative comparison, the visual results show the advantages of the proposed method more clearly. From a visual point of view, except for the proposed SDFormer, CFRL [24], and CACD [55], other methods demonstrate more false detections and missed detections. The proposed approach and CFRL [24] obtain the results closest to the ground reference truth, especially the proposed method, which has the least false detections and missed detections. Therefore, visual BCIs further prove the superiority of the proposed approach.

#### 4.5.2. Results on Data Set #2

**Figure 10:**
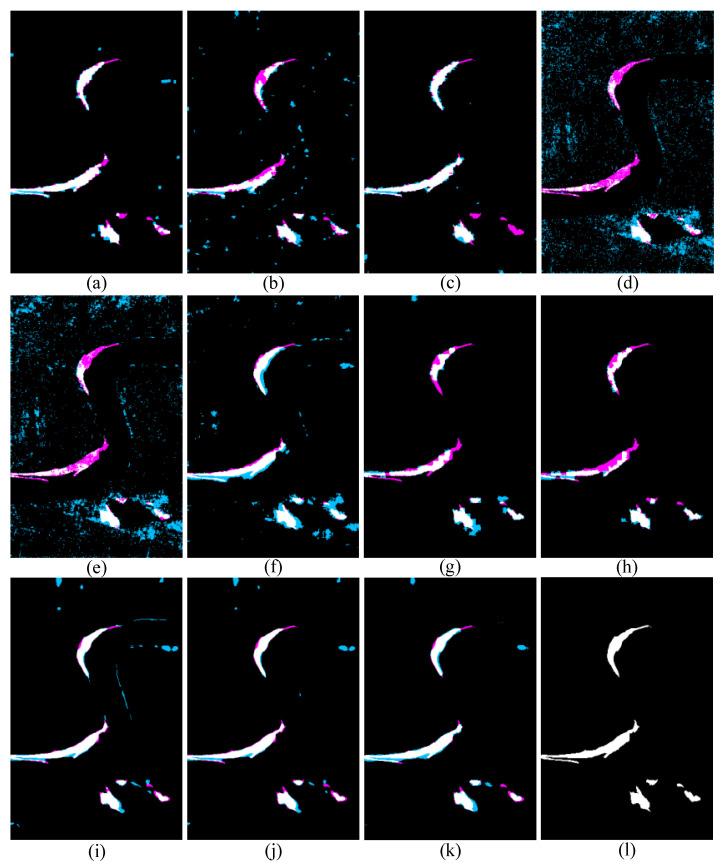
Binary change results of different methods on data set #2: (**a**) IRG-McS [48], (**b**) SCASC [49], (**c**) GIR-MRF [50], (**d**) X-Net [54], (**e**) ACE-Net [54], (**f**) CACD [55], (**g**) SR-GCAE [21], (**h**) AEKAN [23], (**i**) CFRL+Otsu [24], (**j**) CFRL+FLICM [24], (**k**) Ours, and (**l**) Ground truth image. Note: White and black represent TP pixels and TN pixels, respectively; sky blue and magenta represent FP and FN pixels, respectively.

Table 3 and Figure 10 display the quantitative accuracy and visual results of the proposed approach and the comparison methods on data set #2. From the quantitative accuracy comparison results, the proposed SDFormer and CFRL [24] achieve the best accuracy in three indicators, while IRG-McS [48] and GIR-MRF [50] acquire the second-best performance. Moreover, the latest AEKAN [23] achieve the third-best accuracy. Specifically, the proposed SDFormer slightly outperforms CFRL+FLICM [24] in terms of F1 indicator. The proposed method improves the IRG-McS [48] and GIR-MRF [50] by 0.001, 0.021, and 0.021 in terms of OA, KC, and F1, respectively. Compared with the AEKAN [23], the OA, KC, and F1 of the proposed method are improved by 0.005, 0.127, and 0.125, respectively. This also illustrates the advantages of the proposed approach; that is, the proposed SDFormer can perceive the changes between heterogeneous images by intuitively counting the difference information, and the samples obtained based on structural similarity to train SDFormer to better guide the proposed method to achieve effective HCD. Similarly, the visual results comparison also presents the same conclusion. The proposed SDFormer is closer to the ground truth reference than other compared methods and shows fewer false and missed pixels.

#### 4.5.3. Results on Data Set #3

**Figure 11:**
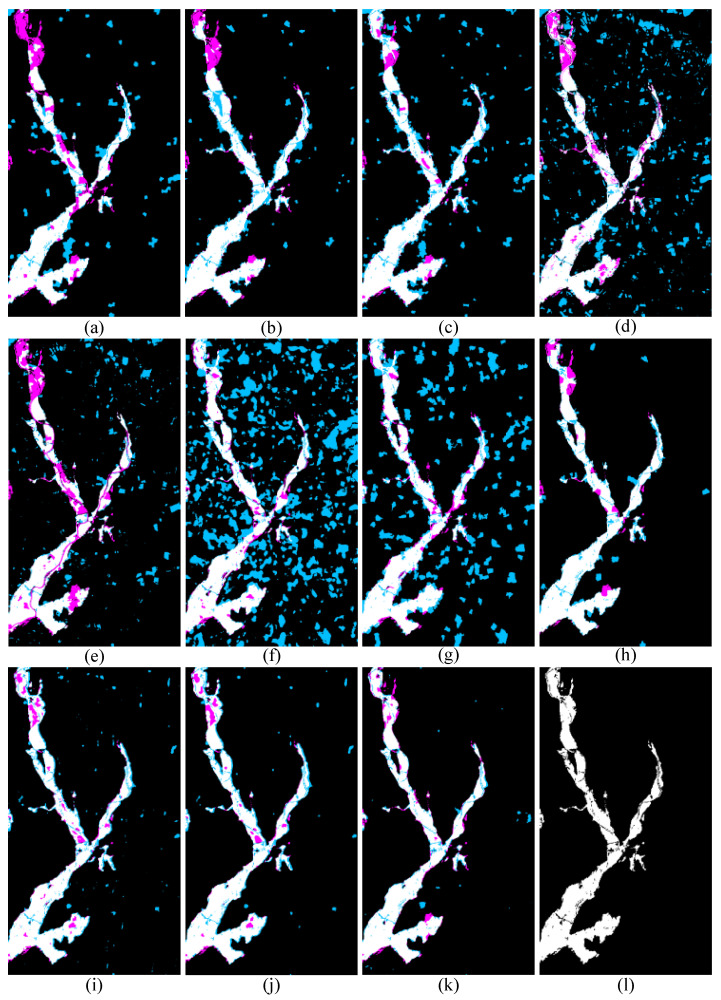
Binary change results of different methods on data set #3: (**a**) IRG-McS [48], (**b**) SCASC [49], (**c**) GIR-MRF [50], (**d**) X-Net [54], (**e**) ACE-Net [54], (**f**) CACD [55], (**g**) SR-GCAE [21], (**h**) AEKAN [23], (**i**) CFRL+Otsu [24], (**j**) CFRL+FLICM [24], (**k**) Ours, and (**l**) Ground truth image. Note: White and black represent TP pixels and TN pixels, respectively; sky blue and magenta represent FP and FN pixels, respectively.

Table 4 and Figure 11 present the quantitative accuracy and visual results of the proposed SDFormer and the comparison approaches on data set #3. The accuracy comparison in Table 4 demonstrates that the proposed SDFormer reaches the best accuracy in all three evaluation indicators, while AEKAN [23] and CFRL+FLICM [24] achieve the second-best and third-best performances. Concretely, compared with the second-best AEKAN [23], the proposed SDFormer obtains improvements of 0.010, 0.042, and 0.035 in OA, KC, and F1, respectively. The proposed SDFormer exceeds the third-best CFRL+FLICM [24] by 0.011, 0.044, and 0.037 in OA, KC, and F1, respectively. The visual result comparison in Figure 11 also presents that the proposed method obtains more accurate and complete detection results. Overall, the experimental results of data set #3 are the same as the results of data sets #1 and #2. It once again proves the effectiveness and superiority of our proposed SDFormer.

## 5. Discussion

In this section, the ablation studies and parameters analysis are performed to further explore the effectiveness of the proposed SDFormer. First, the ablation experiments are conducted on $S^{3}$$G^{2}$ and SDFormer in the proposed method to verify the effectiveness of each part. Second, a series of parameter sensitivity analysis experiments are conducted, including training sample ratio, patch size, and number of super-pixels to analyze the performance of our approach under different parameter settings. The details are as follows.

### 5.1. Ablation Study for Different Components

**Figure 12:**
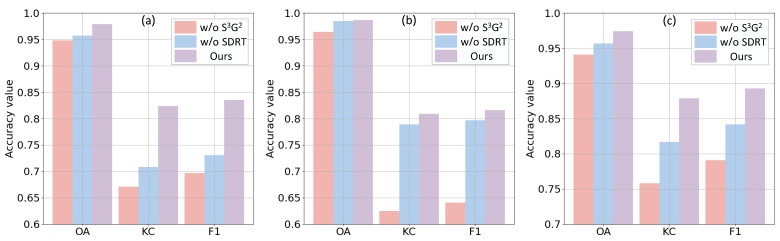
Comparison results of ablation experiments of the proposed SDFormer on three data sets: (**a**) Data set #1; (**b**) Data set #2; (**c**) Data set #3.

In this subsection, in order to test the effectiveness of $S^{3}$$G^{2}$ and SDFormer proposed in this study, these two components are separated from the proposed SDFormer for experiments, respectively. Based on the above settings, as shown in Table 5 and Figure 12, the quantitative and comparative results of ablation experiments on data sets #1–#3 are obtained. From Table 5, it can be found that the performance of the proposed SDFormer is very limited when the proposed $S^{3}$$G^{2}$ or SDFormer is not added, while the performance of the proposed method is significantly improved when these two components are deployed simultaneously, thus achieving the best performance. Specifically, the average accuracy of our method on the three data sets reaches 0.980, 0.837, and 0.848 in terms of OA, KC, and F1, respectively. The average accuracy on the three data sets is reduced by 0.029, 0.152, and 0.138 in terms of OA, KC, and F1, respectively, when $S^{3}$$G^{2}$ is not deployed. Similarly, the average performance on the three data sets is reduced by 0.014, 0.066, and 0.058 in terms of OA, KC, and F1, respectively, when SDFormer is not adopted. Figure 12 presents the impact of different components in the proposed SDFormer on the performance more clearly and intuitively. In general, the above experiments demonstrate the effectiveness of the proposed $S^{3}$$G^{2}$ and SDFormer.

### 5.2. Sensitivity Analysis of Parameters

In this subsection, the three parameters involved in the proposed SDFormer are analyzed to test the impact of these parameters on the proposed approach. Specifically, the proposed method includes three parameters, namely, the training sample ratio, the patch size, and the number of super-pixels. Among them, the ratio of training samples determines the degree of dependence of the proposed method on the sample; the patch size is the analysis unit of our network—a larger patch can provide more information, while a smaller patch may not provide enough information; in the structural similarity-guided sample generation strategy, the number of super-pixels determines the scale of segmentation, which has an important impact on the quality of sample generation. To this end, the parameter analysis experiments on data set #3 are conducted as follows.

#### 5.2.1. Sensitivity Analysis of Ratio of Training Samples

**Figure 13:**
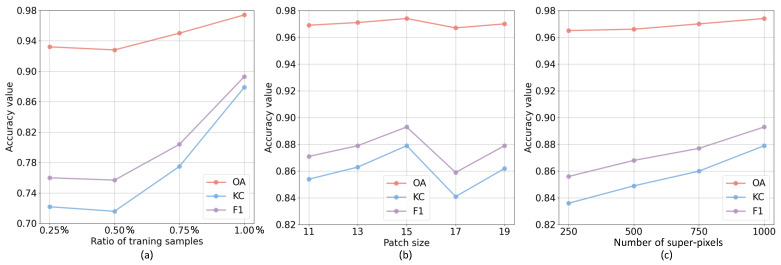
Parameter analysis results of the proposed SDFormer: (**a**) relationship curve between training sample ratio and accuracy; (**b**) relationship curve between patch size and accuracy; (**c**) relationship curve between the number of super-pixels and accuracy.

In order to test the impact of different training ratios on the proposed SDFormer, four different ratios are set for testing, namely: 0.25%, 0.5%, 0.75%, and 1%. Based on this setting, the performance of the proposed SDFormer is acquired under different training sample ratios, and the relationship between them is shown in Figure 13a. Figure 13a shows that as the training sample ratio increases, the Overall Accuracy also shows an upward trend; until the training ratio reaches 1%, OA, KC, and F1 all reach the highest. This illustrates that more training samples are more conducive to improving the detection performance of the proposed SDFormer. In addition, the experiment also shows that the proposed method can still achieve good detection performance when relying on only 0.25% of training samples.

#### 5.2.2. Sensitivity Analysis of Patch Size

The patch size is set to [11, 13, 15, 17, 19] to test the impact of patch size on the detection performance of the proposed SDFormer. The relationship curve between different patch sizes and the three accuracies of OA, KC, and F1 is shown in Figure 13b. We can find that as the patch size gradually increases from 11 to 19, the detection accuracy gradually enhances until it reaches the highest when the patch size is 15, then decreases and then increases. The reason is that a patch that is too large may introduce other objects and introduce inaccurate statistical difference information, resulting in poor accuracy, while a patch that is too small may limit the detection performance due to insufficient information. Therefore, in our proposed SDFormer, choosing an appropriate size is more beneficial to obtain better results in practical application.

#### 5.2.3. Sensitivity Analysis of Number of Super-Pixels

In our proposed SDFormer, a parameter for the number of super-pixels needs to be set, which aims to use structural similarity based on super-pixels to guide the sample generation to select reliable training samples. Reliable training samples are crucial to the proposed approach. In fact, the number of super-pixels determines the scale of super-pixel segmentation. A large number of super-pixels indicates a smaller segmentation scale, while a small number of super-pixels indicates a larger segmentation scale. Therefore, to test the effect of the number of super-pixels on the performance of the proposed SDFormer, the number of super-pixels is set to [250, 500, 750, 1000] to perform sensitivity analysis of this parameter. The experimental results are exhibited in Figure 13c. With the increase in the number of super-pixels, the proposed SDFormer has different degrees of improvement in the three indicators of OA, KC, and F1. This demonstrates that the proposed method has a good performance at different super-pixel scales, and it also presents that our proposed structural similarity-guided sample generation strategy can still obtain reliable training samples when the super-pixel scale is small. Therefore, a relatively moderate segmentation scale is selected to obtain a better performance in practical applications.

## 6. Conclusions

In this paper, a SDFormer with weakly supervised sample generating is proposed for HCD to overcome the limitation due to the lack of reliable labels. In the proposed approach, a structure similarity-guided sample generating strategy is devised, which can acquire reliable pseudo-labels by employing prior information based on differential structure similarity in an iterative generation manner. Moreover, a Statistical Difference representation Transformer is constructed to alleviate the effect of modality differences between heterogeneous images, thereby improving the detection performance in heterogeneous images. Extensive comparative experiments on the three HCD data sets demonstrate the superiority of the proposed approach. Furthermore, sufficient ablation studies and parameter sensitivity analysis experiments validate the effectiveness and reliability of the proposed SDFormer. Nevertheless, our study still has the following two limitations that need further research and improvement. Although the current method can achieve good detection results by relying on less than 1% of samples through weak supervision, its applicability to larger and more complex scenes still needs further testing and exploration.

While the current framework effectively generates pseudo-labels via structural similarity and reduces modality gaps with SDFormer, limitations persist in noisy environments, computational efficiency, and generalization across diverse sensor combinations. Future efforts could focus on integrating uncertainty quantification to enhance pseudo-label robustness, designing lightweight Transformers or dynamic sparse attention for edge deployment, and advancing cross-modal adaptation techniques to improve scalability for multi-source heterogeneous data under limited supervision. 

## Figures and Tables

**Table 1:** Quantitative comparison of AUR and AUP of DIs generated by different methods on data sets #1 to #3.

Methods	Data Set #1		Data Set #2		Data Set #3		Average	
	AUR	AUP	AUR	AUP	AUR	AUP	AUR	AUP
CACD [55]	0.947	0.763	0.978	0.742	0.902	0.459	0.942	0.655
SR-GCAE [21]	0.888	0.339	0.961	0.719	0.957	0.795	0.935	0.618
IRG-McS [48]	0.899	0.643	0.973	0.773	0.946	0.735	0.939	0.717
SCASC [49]	0.886	0.456	0.942	0.594	0.938	0.758	0.922	0.603
GIR-MRF [50]	0.888	0.457	0.932	0.348	0.939	0.759	0.920	0.521
AEKAN [23]	0.950	0.646	0.951	0.712	0.984	0.865	0.962	0.741
CFRL [24]	0.985	0.876	0.994	0.859	0.980	0.773	0.986	0.836
Proposed SDFormer	**0.990**	**0.915**	**0.996**	**0.886**	**0.993**	**0.945**	**0.993**	**0.915**

**Table 2:** Evaluation indicator comparison of different methods on data set #1. In bold are the best results.

Methods	OA	KC	F1
IRG-McS [48]	0.971	0.739	0.754
SCASC [49]	0.947	0.593	0.621
GIR-MRF [50]	0.957	0.674	0.697
X-Net [54]	0.918	0.340	0.443
ACE-Net [54]	0.935	0.549	0.582
CACD [55]	0.975	0.776	0.790
SR-GCAE [21]	0.937	0.546	0.579
AEKAN [23]	0.955	0.660	0.684
CFRL+Otsu [24]	0.973	0.780	0.795
CFRL+FLICM [24]	0.974	0.785	0.799
Ours	**0.979**	**0.824**	**0.835**

**Table 3:** Evaluation indicator comparison of different methods on data set #2. In bold are the best results.

Methods	OA	KC	F1
IRG-McS [48]	0.986	0.788	0.795
SCASC [49]	0.976	0.623	0.636
GIR-MRF [50]	0.986	0.788	0.795
X-Net [54]	0.918	0.232	0.268
ACE-Net [54]	0.928	0.297	0.329
CACD [55]	0.967	0.614	0.630
SR-GCAE [21]	0.981	0.694	0.704
AEKAN [23]	0.982	0.682	0.691
CFRL+Otsu [24]	0.985	0.787	0.795
CFRL+FLICM [24]	0.987	0.809	0.815
Ours	**0.987**	**0.809**	**0.816**

**Table 4:** Evaluation indicator comparison of different methods on data set #3. In bold are the best results.

Methods	OA	KC	F1
IRG-McS [48]	0.936	0.704	0.740
SCASC [49]	0.950	0.776	0.804
GIR-MRF [50]	0.937	0.734	0.770
X-Net [54]	0.909	0.637	0.688
ACE-Net [54]	0.928	0.659	0.701
CACD [55]	0.798	0.417	0.516
SR-GCAE [21]	0.885	0.586	0.649
AEKAN [23]	0.964	0.837	0.858
CFRL+Otsu [24]	0.960	0.822	0.845
CFRL+FLICM [24]	0.963	0.835	0.856
Ours	**0.974**	**0.879**	**0.893**

**Table 5:** Quantitative results of ablation experiments of the proposed SDFormer on data sets #1–#3. The best results are shown in bold.

Methods	Data Set #1			Data Set #2			Data Set #3			Average		
	OA	KC	F1	OA	KC	F1	OA	KC	F1	OA	KC	F1
w/o $S^{3}$$G^{2}$	0.948	0.671	0.697	0.964	0.625	0.641	0.941	0.758	0.791	0.951	0.685	0.710
w/o SDFormer	0.957	0.708	0.731	0.985	0.789	0.797	0.957	0.817	0.842	0.966	0.771	0.790
Ours	**0.979**	**0.824**	**0.835**	**0.987**	**0.809**	**0.816**	**0.974**	**0.879**	**0.893**	**0.980**	**0.837**	**0.848**

## Data Availability

Data are contained within the article.
